# Pulmonary-Resident Memory Lymphocytes: Pivotal Orchestrators of Local Immunity Against Respiratory Infections

**DOI:** 10.3389/fimmu.2021.738955

**Published:** 2021-09-17

**Authors:** Duncan C. Humphries, Richard A. O’Connor, Daniel Larocque, Martine Chabaud-Riou, Kevin Dhaliwal, Vincent Pavot

**Affiliations:** ^1^Centre for Inflammation Research, Queen’s Medical Research Institute, Edinburgh BioQuarter, The University of Edinburgh, Edinburgh, United Kingdom; ^2^Sanofi Pasteur, R&D, Marcy l’Etoile, Lyon, France

**Keywords:** lung, resident memory T cells, resident memory B cells, infection, vaccination, *in situ* optical imaging, EVLP

## Abstract

There is increasing evidence that lung-resident memory T and B cells play a critical role in protecting against respiratory reinfection. With a unique transcriptional and phenotypic profile, resident memory lymphocytes are maintained in a quiescent state, constantly surveying the lung for microbial intruders. Upon reactivation with cognate antigen, these cells provide rapid effector function to enhance immunity and prevent infection. Immunization strategies designed to induce their formation, alongside novel techniques enabling their detection, have the potential to accelerate and transform vaccine development. Despite most data originating from murine studies, this review will discuss recent insights into the generation, maintenance and characterisation of pulmonary resident memory lymphocytes in the context of respiratory infection and vaccination using recent findings from human and non-human primate studies.

## Introduction

Respiratory tract infections remain the leading overall cause of death in developing countries, contributing to 5.4 million deaths annually (1) despite advances in vaccination uptake and technology. Recent evidence has revealed resident memory lymphocyte populations play a key role in the response to reinfection and the development of immune “memory”. Two populations of circulating memory T cells with distinct effector and migratory properties were initially described: central memory T cells (T_CM_) and effector memory T cells (T_EM_). Mechanistic studies in mice demonstrated that T_EM_ were more prevalent in tissues, while T_CM_ were more prevalent in lymph nodes (LN) and persisted following infection (2). T_CM_ access and survey the LN for pathogens using the LN homing receptors C-C chemokine receptor type 7 (CCR7) and CD62-L and have a high proliferative capacity but exhibit low cytotoxicity (2–4). T_EM_ lack or express low levels of CCR7 and CD62-L but express receptors enabling access to peripheral tissues, where upon reencounter with cognate antigen they rapidly exhibit high cytotoxicity (2–4). This concept has since been refined after it was found that T_EM_ are largely excluded from tissue and are restricted to the spleen and intravascular compartment (4). A novel subset of memory T cells that share similarities to both T_CM_ and T_EM_, termed peripheral memory T cells (T_PM_), have been identified as the predominant subset that re-circulate between blood and peripheral tissues (4). It is now recognised that additional subset designations exist, and memory T cells fall on a continuum, rather than rigid subsets, based on their localisation, trafficking, metabolism, longevity, and phenotypic characteristics (5).

During the last decade, a memory T cell subgroup found to reside long-term in tissues without recirculating in blood has been identified. Lacking CD62-L and CCR7, resident memory T cells (T_RM_) function as a first line of adaptive immune defence against subsequent re-infection and constitute the majority of T cells within the lung (5, 6). Lung-resident memory B cells (B_RM_) have also been recently recognised for their critical role in immunity to respiratory infection (7). Maintained in a quiescent state, B_RM_ await secondary challenge where they accelerate secondary B cell responses.

Humans frequently develop respiratory infections throughout life and the current global coronavirus disease 2019 (COVID-19) pandemic has highlighted the need to develop and distribute effective vaccines to prevent/reduce key infectious respiratory diseases. Therefore, the development of new vaccines (e.g. COVID-19, respiratory syncytial virus, Middle East Respiratory Syndrome coronavirus) and the improvement of existing vaccines (e.g. tuberculosis, pertussis, pneumococcal and influenza) able to induce long-lasting immunity and prevent such diseases is urgently needed. The role of T_RM_ and B_RM_ in the control of respiratory infections has been highlighted recently in human and animal models (7, 8). Vaccination strategies that enhance either pre-existing memory T and B cells or promote the establishment of new antigen-specific T_RM_/B_RM_ populations and their maintenance, alongside novel techniques for their *in situ* detection and functional characterisation, will be important tools for developing vaccines that provide long-lasting immunity against heterosubtypic infection. Here, we discuss the current knowledge of pulmonary T_RM_ and B_RM_ in human and animal models in the context of infection, highlighting knowledge gaps and opportunities in vaccine development.

## Formation and Maintenance of Pulmonary T_RM_ and B_RM_

### Generation of Pulmonary T_RM_

**Figure 1:**
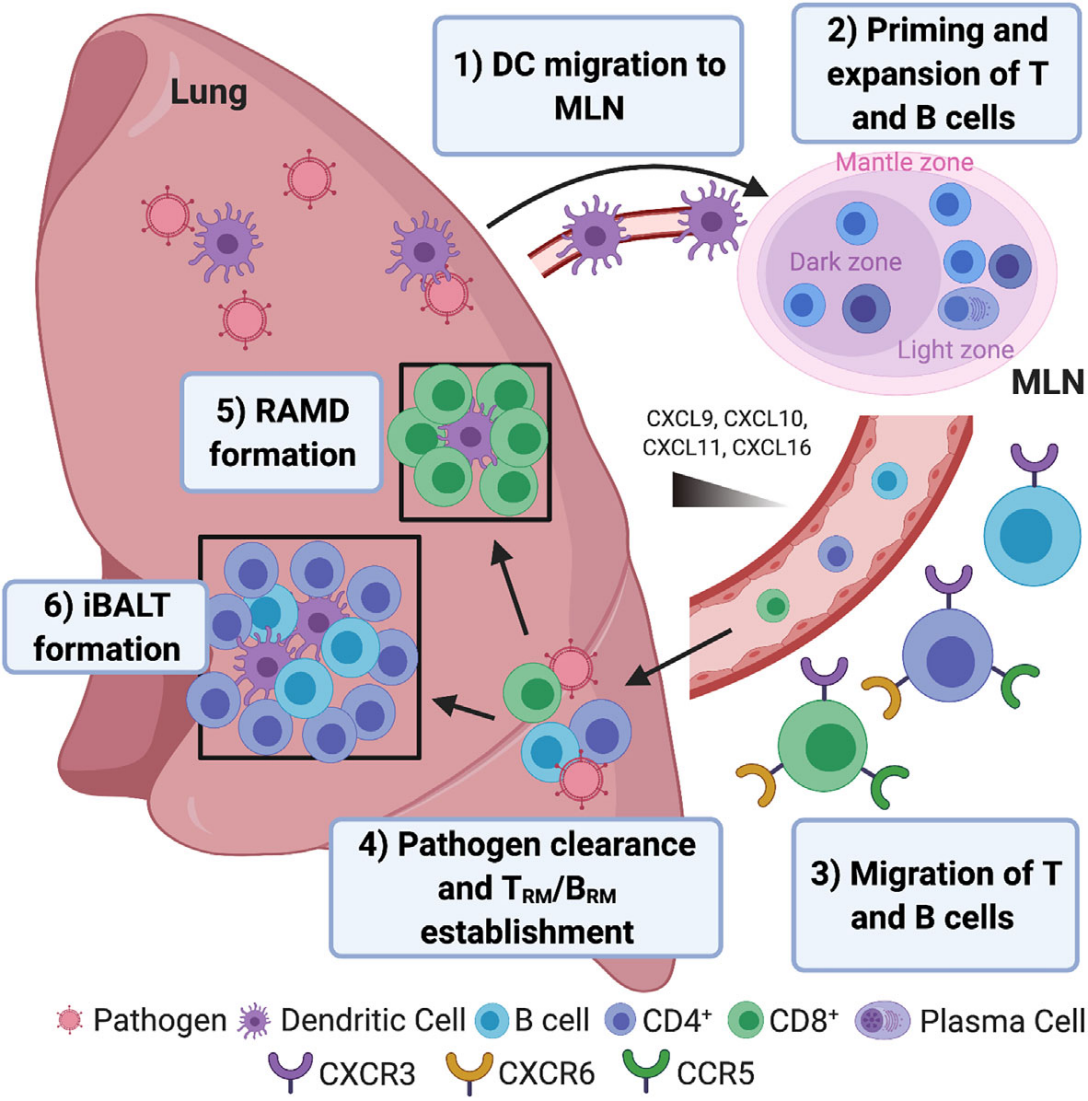
Pulmonary resident memory lymphocyte formation. **1)** Inhaled respiratory pathogen (viral/bacterial) antigens are processed and presented by dendritic cells (DCs) that migrate to the mediastinal lymph node (MLN). **2)** DCs prime naïve CD4^+^ and CD8^+^ T cells in MLN with cognate antigen expressed on MHC II and MHC I, respectively, resulting in T cell proliferation. B cells interact with cognate CD4^+^ T cells at the border between the B and T cell zones within Germinal Centres (GCs), becoming short-lived, antibody-secreting plasma cells or early memory B cells (IgM^+^) or enter the GC and undergo somatic hypermutation and isotype switching, with low affinity B cells differentiating into memory cells to ensure a degree of poly-reactivity. High affinity B cells differentiate into long-lived plasma cells and migrate to the bone marrow where they secrete antibodies for decades (9). **3)** Stimulation within the MLN leads to the expression of chemokine receptors CXCR3, CXCR6 and CCR5 that enable T cell trafficking to the lung and airways following CXCL9/CXCL10/CXCL11/CXCL16 chemokine gradients. Pulmonary epithelial cells, DCs and macrophages secrete CCR5 and CXCR3 binding chemokines following respiratory infection (10). The CXCR6 ligand, CXCL16, is also expressed by lung bronchial epithelial cells and may also play a role in T cell homing (11). Memory B cells also migrate to the infected lung, following interferon-inducible chemokines CXCL9, CXCL10 and CXCL11 *via* CXCR3 (12, 13) where they are strategically located for subsequent reinfection. **4)** Once entered the lung, effector T cells and short-lived plasma cells help clear infection and undergo apoptosis. A minority of effector T cells differentiate into pulmonary-resident memory T cells (T_RM_). IgM^+^ pulmonary-resident memory B cells (B_RM_) seed the lung early after infection, followed by isotype-switched B_RM_ (7). **5)** CD8^+^ T_RM_ accumulate and self-renew in areas undergoing tissue regeneration following infection known as repair-associated memory depots (RAMD) where they seed airway CD8^+^ T_RM_, which are ideally located for pathogen clearance in the case of reinfection. **6)** CD4^+^ T_RM_ and B_RM_ reside within GCs of inducible bronchus-associated lymphoid tissue (iBALT). Associated with prolonged persistence of antigens, iBALT GCs in infected lungs serve as sites for exaggerated B cell proliferation and cross-reactive clonal selection of plasma cells/memory progenitors following B cell/CD4^+^ T_RM_ interactions (14).

Professional antigen presenting cells (APCs) including dendritic cells (DCs) are key regulators of innate and adaptive immune responses. During primary viral/bacterial respiratory infection, lung-resident DCs process and present the pathogen’s antigens and migrate to the mediastinal lymph node (MLN) to prime naïve T cells and stimulate their proliferation (**Figure 1**). Migratory lung DCs within the MLN imprint T cell lung homing through site-specific surface molecular signatures (15, 16) and help influence pulmonary T_RM_ generation. In human and humanized mice, pulmonary CD1c^+^ and CD141^+^ DCs have both been shown to present viral antigens, however only CD1c^+^ DCs drive the expression of CD103 (a key marker of T_RM_ – see “Phenotypic Characterisation”) on both naïve and memory CD8^+^ T cells (17). Multiple chemokine receptors involved in lung trafficking are expressed by T_RM_ including C-X-C Motif Chemokine Receptor 3 (CXCR3), CXCR6 and CCR5 (11, 18–20). Although no specific combination of homing markers have been identified for pulmonary T_RM_, CD4^+^ are likely recruited to the airway during Respiratory Syncytial Virus (RSV) infection in human *via* C-X-C motif chemokine 10 (CXCL10 - the ligand for CXCR3), as chemokine levels correlated with activated CD4^+^ T cell recruitment in bronchoalveolar lavage (BAL) (18).

Shortly after activation in the MLN, effector T cells migrate to the lungs and contribute towards pathogen clearance. The majority of pathogen-specific T cells then undergo apoptosis, however a minority differentiate into T_RM_ in response to environmental cues (21), with the number of T cells persisting in the lung following infection correlating with the efficiency of T_RM_ differentiation (22).

Effector T cells entering the lung express sphingosine-1-phosphate receptor (S1PR1), sensing increasing sphingosine-1-phosphate (S1P) gradients in blood and lymph, leading to tissue egress (10). S1PR1 expression is regulated by local cytokine-induced transcriptional downregulation and early activation marker CD69-mediated post-transcriptional antagonism (10). CD69 is a cell-surface receptor that is rapidly and transiently expressed on all recently activated T cells. Induction of the membrane‐bound type II C‐lectin receptor CD69 by antigen stimulation and inflammatory cytokine exposure leads to downregulation of S1PR1, which when combined with inflammation-induced chemotactic signalling, supports effector T cell retention and T_RM_ generation (10, 23). Transition of recruited effector T cells to T_RM_ in murine lung requires simultaneous tissue damage and T cell receptor (TCR) activation by pulmonary cognate antigen encounter (24–27). Overlapping TCR genes from human T_RM_ and non-T_RM_ indicate that environment, rather than epitope specificity, drives T_RM_ formation (19). Antigen-dependent cross-competition however does promote T_RM_ formation, with effector T cells recognising antigen presented by infected tissue cells preferentially entering the local T_RM_ pool (28). Although demonstrated in murine skin, it is possible the same rules also apply to the lung. Naïve T cells in LNs may also be epigenetically preconditioned during steady state conditions by migratory DCs to differentiate into T_RM_ upon exposure to cognate antigen (29). Dependent on DC-driven, transforming growth factor β (TGF-β), altering local or systemic TGF-β activity prior to vaccination may help promote T_RM_ formation (29).

Once established, T_RM_ remain lung-resident and contribute towards immunosurveillance and homeostasis (6). Maintained in a quiescent state, human transplant studies have demonstrated donor CD4^+^ and CD8^+^ T_RM_ to persist in the lungs for over 15 months, with single cell transcriptome analysis confirming *de novo* T_RM_ generation *via* the identification of a “mature T_RM_” and an immature “T_RM_-like” population that gradually acquire T_RM_ markers (CD69, CD103 and CD49a) over time (30, 31). Pulmonary CD8^+^ T_RM_ are however more short-lived than those found in other tissues such as the skin and intestine (32, 33). As microbes are constantly being inhaled, the limited longevity of pulmonary CD8^+^ T_RM_ may provide a mechanism for avoiding unnecessary inflammation and pathogenesis in this tissue (34).

### Gene Regulation in T_RM_

In human, Notch signalling alongside low levels of T-bet and Eomesodermin (EOMES) are required for the development and maintenance of CD4^+^/CD8^+^ T_RM_, with Notch regulating T_RM_ metabolic programs (11, 20). Human pulmonary CD8^+^ T_RM_ display elevated levels of the transcription factors Hobit (encoded by the gene *ZNF683*) and Runx3, that may be involved in T_RM_ generation and/or maintenance (30). Interestingly, despite showing elevated mRNA levels, Hobit protein expression was reported absent in human CD4^+^ T_RM_, suggesting differences between CD4^+^/CD8^+^ T_RM_ formation/maintenance (11).

Heterogeneity in effector function and phenotype is evident within T_RM_ populations, particularly within CD4^+^ T_RM_ (19). Transcriptome profiling of human lung CD69^+^ T_RM_ has revealed the differential expression of 31 core genes associated with migration, adhesion and regulatory molecules when compared to CD69^-^ subsets (19). This transcriptional profile is conserved across CD4^+^/CD8^+^ CD69^+^ lineages as well as tissues (19). Pulmonary T_RM_ exhibit high transcript levels for genes encoding for several chemokine receptors, pro-inflammatory cytokines and cytotoxic mediators, enabling them to be recruited and retained within the lung and undergo rapid, polyfunctional responses (11, 20). T_RM_ respond rapidly with effector functions, however, expression of regulatory genes (e.g. cytotoxic T-lymphocyte-associated protein 4 [CTLA4] and B-and T-lymphocyte attenuator 4 [BTLA4]) in CD8^+^ T_RM_ may present a safety mechanism to minimise aberrant activation and associated inflammation/tissue damage (20).

### Generation of Pulmonary B_RM_

Human antigen-experienced lungs are enriched with B cells containing a resident memory phenotype (35). As human and non-human primate (NHP) B_RM_ data are limited, most findings are derived from mouse studies. During primary respiratory infection, naïve B cells, primed by either free antigen or antigen delivered by subcapsular sinus (SCS) macrophages (36), interact with cognate CD4^+^ T cells at the T-B border within the MLN (9, 37). Following initial proliferation at the outer follicles, B cells may differentiate into extrafollicular short-lived plasma cells, early (germinal centre (GC)-independent) memory cells or proliferate to form the GC (**Figure 1**). Following somatic hypermutation, B cells can exit as long-lived plasma cells, migrating to the bone marrow where they secrete antibodies for decades, or memory B cells (9, 37). Having migrated to the lungs to participate in pathogen clearance, most of the responding B cells undergo apoptosis, leaving a few resting memory cells in the respiratory tract and lymphoid organs where they wait for the same antigen.

Murine parabiosis studies have demonstrated B_RM_ generation requires local antigen encounter and is dependent on early CD40-interactions with T cells (7). Once established, B_RM_ remain lung resident due to expression of CD69 (7). Here they undergo metabolic reprogramming, switching from anabolic to catabolic pathways to reduce their requirement for high levels of cytokines for their maintenance (37). In mice, B_RM_ are quiescent and long-lived, maintained from precursors within persisting GCs in areas known as inducible bronchus-associated lymphoid tissue (iBALT) (14), however B_RM_ have also been detected in the absence of iBALT (39). Established one week after influenza infection, murine pulmonary B_RM_ have been demonstrated to be phenotypically and functionally distinct from their systemic counterparts (7).

### Gene Regulation in B_RM_

Few studies have investigated gene regulation in pulmonary B_RM_, particularly in humans. Although the possibility of a “master transcription factor” for B_RM_ generation has been suggested, no unique transcription factor has been identified so far (9). Increased expression of the transcription factors Bach2, KLF2, ZBTB32, ABF1 and STAT5 are associated with B_RM_ formation in mice, however their exact roles are yet to be understood (9, 40). The transcriptional regulation of pulmonary B_RM_ differentiation is likely to be unique – understanding these transcription factors may help identify methods for modulating their formation (41).

## Phenotypic Characterisation

### Human and Non-Human Primate T_RM_ Markers

Due to their similarities to human, NHPs provide an invaluable tool for investigating host response to respiratory infection and vaccination. Although heterogenous within the lung, human and NHP T_RM_ are phenotypically distinct from T_CM_ and T_EM_ and are primarily identified by the high expression of the C-type lectin receptor CD69, and integrins CD103 and CD49a (30). The transmembrane CD69 is a key marker of pulmonary T_RM_, distinguishing memory T cells in tissue from those in circulation (19), however murine evidence suggests its expression is not essential for the establishment and maintenance of T_RM_ in the lung (25, 42, 43). Although considered as an early activation marker for TCR signalling, T_RM_ CD69 expression is not associated with markers of recent activation and appears to be a function of previous antigen exposure (19).

Preferentially expressed on CD8^+^ T_RM_ compared to CD4^+^, CD103 promotes adherence to E-cadherin, an adhesion molecule expressed by epithelial cells (22, 30, 44). CD103 expression is driven by membrane-bound TGF-β (mediated by IL-10) on APCs (CD1c^+^ DCs and monocytes) (17, 45) and is thought to contribute towards initial recruitment and persistence of CD8^+^ T_RM_ to aide surveillance rather than long-term maintenance (42). CD49a, expressed by both CD4^+^ and CD8^+^ T_RM_, is an integrin specific to collagen IV that facilitates locomotion for surveillance and is essential for T_RM_ survival by limiting apoptosis following ligand engagement (42). Other recognised surface markers of pulmonary T_RM_ are outlined in **Table 1** - understanding the full function of these markers, whether they represent different subsets/maturation states and whether they are pathogen-dependent remains to be determined.

**Table 1:** Human and Non-Human Primate Surface Marker Expression on Pulmonary T_RM_.

Surface Marker	Cell Type	Function	Pathogen/Condition Studied	Species + References
CD69	CD4^+^ T_RM_	Tissue retention	Lung Donation, *Mtb, RSV, Influenza*	Human (18, 19, 22, 24, 30, 46)
				NHP (44)
	CD8^+^ T_RM_		Lung Donation*, Mtb, RSV, Influenza*	Human (8, 18, 19, 22, 24, 30, 45, 46)
				NHP (21, 44, 45)
CD103 (αE integrin)	CD4^+^ T_RM_	Adhesion to E-cadherin, initial recruitment, facilitates persistence and surveillance	Lung Donation, *Mtb*, biopsy, *RSV, Influenza*	Human (11, 18, 19, 22, 24, 30, 46)
	CD8^+^ T_RM_		Lung Donation, *Mtb*, biopsy, *RSV, Influenza*	Human (8, 18, 19, 22, 24, 30, 45, 46)
				NHP (21, 45)
CD49a (α_1_β_1_ integrin/VLA-1)	CD4^+^ T_RM_	Adhesion to Collagen IV, limits apoptosis, facilitates locomotion for surveillance	Lung Donation, *Mtb*	Human (19, 24, 30)
	CD8^+^ T_RM_		Lung Donation, *Mtb*	Human (19, 24, 30)
CD49d (α_4_β_1_ integrin/VLA-4)	CD4^+^ T_RM_	Adhesion to Fibronectin	*Mtb*	Human (24, 47)
CD101	CD4^+^ T_RM_	Inhibits T cell activation, proliferation	Lung Donation	Human (30)
	CD8^+^ T_RM_		Lung Donation, *Mtb*	Human (19, 24, 30)
PD-1 (CD279)	CD4^+^ T_RM_	Immune checkpoint and T cell exhaustion marker (prevent aberrant activation)	Lung Donation, *Mtb*	Human (19, 30)
				NHP (48)
	CD8^+^ T_RM_		*Influenza*	Human (19, 30, 46)
				NHP (44)
CXCR3	CD4^+^ T_RM_	Chemokine receptor	*Mtb, RSV*	Human (11, 18)
	CD8^+^ T_RM_		Biopsy	Human (20)
CXCR6	CD4^+^ T_RM_	Chemokine receptor	Lung Donation, biopsy	Human (11, 19, 46)
	CD8^+^ T_RM_		Lung Donation, biopsy	Human (19, 20, 46)
CCR5	CD4^+^ T_RM_	Chemokine receptor	Lung Donation/cancer lobectomy, *Mtb*	Human (11, 18, 46)
	CD8^+^ T_RM_		*Mtb*	Human (46)
CCR6	CD8^+^ T_RM_	Chemokine Receptor	Lung Resection	Human (20, 49)
CD44	CD8^+^ T_RM_	Leukocyte rolling and adhesion	*Mtb, influenza*	Human (24)
CD28/CD28H	CD8^+^ T_RM_	T cell activation	Lung Resection	Human (11, 49)
CD45RO	CD4^+^ T_RM_	Memory T cell marker	*Influenza*	Human (33)
	CD8^+^ T_RM_		Lung donation, *Influenza*	Human (22, 33)
CD45RA^-^	CD4^+^ T_RM_	Naïve T cell marker	Lung Donation	Human (19, 30)
	CD8^+^ T_RM_		Lung Donation	Human (19, 30)

Multiple markers relating to adhesion/migration/activation are specifically upregulated on lung T_RM_. Other naive/effector/memory markers help distinguish memory T cells from regular effector T cells (e.g. CD45RA and CD45RO). Mtb, Mycobacterium tuberculosis; RSV, Respiratory Syncytial virus.

### B_RM_ Markers

Although no specific marker of B_RM_ residency has been described, pulmonary B_RM_ are phenotypically distinct from their systemic memory and non-memory counterparts (7) – see **Table 2**. As well as lacking CD62-L, murine pulmonary B_RM_ express markers associated with T_RM_, such as CD69, CXCR3 and CD44, which retain B_RM_ within the lung (7, 12, 35, 39). CD69 has also been found on human pulmonary B_RM_ (35). Whether other markers found in mice are also expressed on human and NHP pulmonary B_RM_ requires further investigation.

**Table 2:** Surface Marker Expression of Human/Mouse Pulmonary B_RM_.

Surface Marker	Function	Pathogen/Condition Studied	Species + References
CD38	Cell adhesion	*Influenza*	Mouse (12)
CD80	GC-matured memory marker	*Influenza, Pneumococcus*	Mouse (7, 12, 35, 39)
CD27	Post- activation marker, memory B cell marker	Healthy lung resection/lobectomy	Human (35)
CD73	GC-matured memory marker	*Influenza, Pneumococcus*	Mouse (12, 35, 39)
PD-L2 (CD273)	GC-matured memory marker	*Influenza, Pneumococcus*	Mouse (7, 12, 35, 39)
CD20	B cell differentiation	*Pneumococcus*	Mouse (39)
CD69	Tissue retention	Healthy lung resection/lobectomy*, Influenza, Pneumococcus*	Human (35)
			Mouse (7, 12, 35, 39)
CD44	Leukocyte rolling and adhesion	*Pneumococcus*	Mouse (35, 39)
CD11a	Integrin, cell adhesion	*Pneumococcus*	Mouse (35)
CXCR3	Chemokine receptor	*Influenza*	Mouse (7, 12)

B_RM_ surface markers are mostly associated with activation, GC-maturation and tissue homing and share some similarities with T_RM_.

Functional studies have revealed B_RM_ established early after murine influenza infection are positive for immunoglobulin M (IgM^+^) which are later followed by isotype-switched B_RM_ (7). Following murine *pneumococcal* infection, the majority of isotype-switched B_RM_ are IgG^+^, with a small fraction IgA^+^ (35). The majority of B_RM_ found in healthy human lung are also isotype-switched (35).

## Anatomical Location

**Figure 2:**
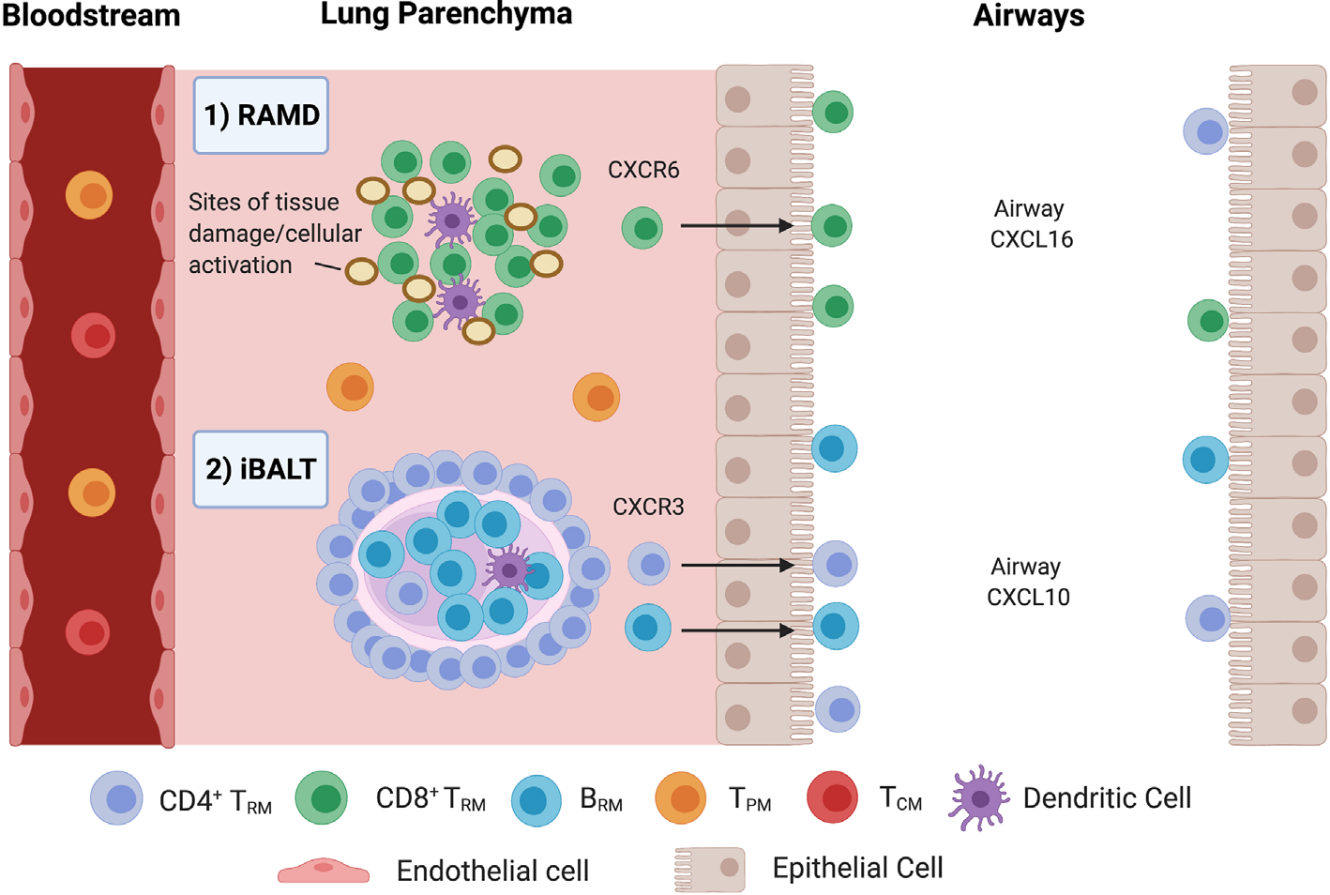
Compartmentalisation of Pulmonary T_RM_ and B_RM_. **1)** CD8^+^ T_RM_ are maintained in repair-associated memory depots (RAMDs) located in peribronchiolar foci in areas previously damaged from primary infection. RAMDs can be identified *via* the presence of cytokeratin-expressing cell aggregates which contain distal airway stem cells that help reconstruct damaged lung tissue (10). Murine evidence suggests interstitial CD8^+^ T_RM_ are primarily maintained by a process of homeostatic proliferation and seed airway T_RM_, driven by CXCR6 in response to airway CXCL16 (38). **2)** CD4^+^ T_RM_ surround B_RM_ cell follicles in iBALT located within the pulmonary parenchyma, where prolonged antigen persistence enhances CD4^+^ T_RM_/B_RM_ formation. Just like RAMDs, iBALT requires tissue damage/inflammation for their establishment. CD4^+^ T_RM_ are then recruited to the alveolar space *via* CXCL10/CXCR3.

T_RM_/B_RM_ persist at sites of previous antigen encounter (13). CD8^+^CD103^+^ T_RM_ are found at higher frequencies in the airway than in parenchyma due to adhesion to epithelial E-cadherin, making them ideally located to respond to reinfection (30). Murine CD8^+^ T_RM_ reside and self-renew in peribronchiolar foci in areas undergoing tissue remodelling, known as repair-associated memory depots (RAMDs) (25, 38) (**Figure 2**). Tissue damage is a requirement for RAMDs (25) and may have implications for vaccine design and delivery. The existence of human RAMDs containing CD8^+^ T_RM_ remains to be confirmed.

CD4^+^ T_RM_ and B_RM_ are located and maintained around the small airways within iBALT located within the parenchyma (13, 14, 25, 30) (**Figure 2**). iBALT contain GCs that serve as sites for B cell selection and maturation following murine influenza infection (50), generating cross-reactive memory B cells to ensure heterosubtypic humoral protection (14). Formation is associated with inflammation (44) and prolonged antigen persistence (14). B_RM_ have also been observed in non-lymphoid areas below the airway epithelium and airways (13) as well as lungs of *pneumococcal*-recovered mice lacking iBALT, indicating that they are components of histologically unremarkable lungs that may not require iBALT for their maintenance (35, 39).

## Function

### T_RM_ Response to Viral Infection

Growing evidence indicates that virus-specific T cells resident along the respiratory tract are highly effective at providing potent and rapid protection against inhaled pathogens. In human influenza infection, CD8^+^ T_RM_ have been shown to recognise the internal, conserved proteins of the virus whereas CD4^+^ T_RM_ recognise both internal and external proteins, with both cell types contributing towards heterosubtypic protection (33). CD8^+^ T_RM_ have been shown to be cross-reactive against three influenza strains (51), with single cell sequencing revealing diverse TCR profiles “capable of recognising newly emerging viral escape variants” (22).

Influenza-specific CD8^+^ T_RM_ have a low activation requirement, requiring only cognate antigen in the absence of helper cell-derived signals (52). Once stimulated, they are highly proliferative, producing polyfunctional progeny (producing ≥2 cytokines – IFN-*γ*, TNF, Granzyme B and IL-2) with effector function superior even to their parent population (22, 44). Polyfunctional T_RM_ offer enhanced protection by producing higher levels of cytokines whilst simultaneously driving effector responses (53) - activated CD8^+^ T_RM_ exert their cytotoxic function to kill infected cells (10) whilst CD4^+^ T_RM_ interact with B cells in iBALT to generate new neutralising antibodies (14, 54). A newly identified, long-lived CD4^+^ T resident helper (T_RH_) population with functional and phenotypical similarities to lymphoid T follicular helper cells (T_FH_) has also been described following murine influenza infection. Residing within iBALT, T_RH_ are tightly localised with B_RM_ to support local antibody production following reinfection (55).

In an experimental human RSV infection model, the abundance of RSV-specific, pulmonary CD8^+^ T_RM_ before infection was associated with reduced symptoms and viral load, implying that CD8^+^ T_RM_ can confer protection against severe respiratory viral disease when humoral immunity is overcome (8). RSV-specific CD8^+^ T_RM_ displayed phenotypic changes representative of advanced differentiation, with downregulation of both co-stimulatory and cytotoxicity markers, suggesting cells can respond rapidly to reinfection, but function is restricted to minimise excessive tissue damage (8).

RSV infection in African Green Monkeys (AGM) also induced virus-specific airway CD8^+^ T_RM_ capable of reducing viral titres, however failed to induce robust CD4^+^ T_RM_ and humoral responses (21). Previously protective RSV-candidate vaccines in AGM induced a strong T cell response, whilst those eliciting a strong neutralisation antibody response without detectable T cell response were not as effective (56). Similar to influenza, CD8^+^ T_RM_ recognise internal proteins of RSV, whilst CD4^+^ T_RM_ recognise external proteins (18). RSV-induced immunopathology relates to a dysregulated T cell response – RSV-specific memory CD8^+^ T cells in blood display little evidence of multiple cytokine production unlike those seen against influenza (8, 18). CD8^+^ T_RM_ however appear to be more polyfunctional, generating IFN-*γ*, IL-2 and TNF (21), however fail to undergo proliferation when activated and express reduced cytotoxicity markers compared to peripheral memory cells (8).

### T_RM_ Reponses to Bacterial Infection

Activated CD4^+^ and CD8^+^ T_RM_ have been identified in the lungs of patients infected with *Mycobacterium tuberculosis* (*Mtb*), where they help limit intracellular macrophage *Mtb* replication (46). T_RM_ were polyfunctional, expressing IFN-*γ*, TNF ± IL-2, and exhibited a highly cytotoxic profile, with CD4^+^ T_RM_ appearing more polyfunctional than CD8^+^ T_RM_ (46). CD49d is upregulated on airway CD4^+^ T_RM_ and optimises the localisation of human *Mtb* **-**specific recall responses (47). *Mtb* infection in macaques drives a cellular T helper 1 (T_H_1) and humoral response, without protective efficacy, however repeated pulmonary Bacillus Calmette-Guérin (BCG) delivery was shown to induce polyfunctional, T_H_17 CD4^+^ T_RM_, leading to airway IgA secretions in BAL (48), presumably through the generation of B_RM_. Interstitial CD4^+^ depletion with *simian immunodeficiency virus* (SIV) following *Mtb* infection identified CD4^+^ T_RM_ (57), proliferating CD8^+^ memory T cells (T_CM_, T_EM_ and likely T_RM_) and B cells within iBALT (58) as critical for suppressing latent *Mtb* reactivation.

T_H_17 CD4^+^ T_RM_ are also critical in protecting against murine nasal *Bordetella pertussis* (Bp) colonization (59). Although both capable of protecting against Bp lung infection, whole cell Bp vaccine, unlike the acellular vaccine, induced nasal IL-17-producing CD103^+^ CD4^+^ T_RM_ (similar to natural Bp infection) that recruited neutrophils to enhance bacterial clearance.

### T_RM_ Bystander Effect

Lung T_RM_ also display “innate-like” behaviour, amplifying inflammation following noncognate bacterial infection. APC-derived IL-12/IL-18 activated virus-specific CD8^+^ T_RM_ within the lung parenchyma, leading to the rapid synthesis of IFN-*γ*. This “bystander activation” boosted neutrophil recruitment to improve bacterial clearance. Despite being performed in mice, the authors demonstrated *in vitro* that human CD8^+^ T_RM_ similarly synthesise IFN-*γ* in response to IL-12/IL-18 (60).

### B_RM_ Response to Viral and Bacterial Infections

Alongside long-lived antibody-secreting plasma cells, B_RM_ contribute towards the protective humoral immune response to pulmonary reinfection (12). The presence of B_RM_ is a common feature of antigen‐experienced lungs and is important for acquired immunity (7). B cells in the airways secrete antibodies that act both locally and at mucosal surfaces. These antibodies, predominantly IgM and IgA, bind to glandular epithelial and mucosal surfaces to promote pathogen clearance (61). B cells activated in respiratory lymphoid tissue also differentiate into IgA‐secreting plasma cells that predominantly act in the airway. Current knowledge of B cell homing and class switching in the airway remains limited.

Murine parabiosis/adoptive transfer/depletion studies have demonstrated the protective role played by B_RM_ in response to both viral (7, 12, 54) and bacterial lung infection (35). B_RM_ provide rapid antibody-secreting cells (ASC), producing a range of class switched neutralising antibodies (7, 12). Lung B_RM_ produce greater numbers of ASC than splenic memory cells following exposure to drifted virus, indicative of heterosubtypic protection (14). Cross-neutralising antibodies to conserved, internal influenza proteins provide heterosubtypic protection (14, 54). Although IgA is more effective than IgG at preventing upper respiratory infection, in combination they achieve maximal neutralising activity against influenza in mice (12). Following murine *pneumococcal* infection, B_RM_ contribute towards bacterial clearance by rapidly secreting cross-reactive antibodies, even when reactivated by a serotype-mismatched strain (35). In macaques, iBALT persistence is associated with reduced *Mtb* reactivation due to enhanced B-cell and humoral immunity (58). B_RM_ are also potent APC, binding and endocytosing antigen *via* their BCR to increase peptide/MHC II presentation and further enhance CD4^+^/B cell responses (9, 13).

## Loss of Pulmonary Protection

Pulmonary immunity to respiratory pathogens wanes over time, meaning individuals are susceptible to recurrent infections throughout their lifetime. Although antigen drift may contribute to loss of protection, the gradual loss of pulmonary CD8^+^ T_RM_ is a major contributor (32). Murine lung CD8^+^ T_RM_ are less durable than those found in skin due to an increased susceptibility to apoptosis (32), and have been shown to undergo “retrograde migration” to the MLN where they provide longer-lived regional memory (62). Loss of RAMDs due to tissue repair correlated with a decline in CD8^+^ T_RM_ number in mice (10, 25), whilst in humans iBALT diminishes with age (6) which may explain why older age groups are more susceptible to respiratory infection due to a reduced ability to mount CD4^+^ T_RM_/B_RM_ responses.

## Immunopathology

Although T_RM_-driven immunopathology has been described in other tissues (63), less is known regarding pulmonary T_RM_. Moderate-severe asthma patients display increased numbers of CD4^+^CD103^+^ T_RM_ in their airways (64) and vaccine-enhanced disease in children with formalin-inactivated RSV is driven by T_H_2 CD4^+^ memory cells that induce excessive inflammation (65). Exacerbations of pulmonary pathology following RSV infection have also been linked to iBALT which stimulate increased, yet detrimental, immune responses (66). CD8^+^ T_RM_ may impact gas exchange *via* the presence of RAMD or through inflammation induced by bystander activation (60). *In vitro*, CD8^+^ T cells damage non-infected epithelial cells during influenza infection through TNF and IFN-*γ* release (67). Although the detrimental effect due to T_RM_/B_RM_ has not been demonstrated *in vivo*, T_RM_/B_RM_ formation may not always be beneficial if accompanied by another immune cell influx such as that found following acute infections.

## Vaccination Strategies to Promote T_RM_

The presence of pathogen-specific T_RM_ cells in the lungs has been shown to correlate with protection in human and animal models. It has therefore been proposed that T_RM_ represent one of several immune mechanisms that should be harnessed together for optimal vaccine-mediated protection. A better understanding of how lung T_RM_ are generated and maintained is required for optimal vaccine development. Vaccination strategies to promote T_RM_ have been successfully demonstrated in mouse models, including engineered biomaterials that modulate antigen delivery and retention time, adjuvant combinations, viral vectors and virus-like particles, as well as direct APC targeting (68), however studies in human and NHP are limited.

In mice and human, inactivated influenza vaccines induce systemic humoral responses but fail to induce T cell immunity in the lungs (33, 69, 70). Intranasal live-attenuated influenza virus vaccines however generate mucosal IgA, lung CD4^+^ T_RM_ and virus-specific CD8^+^ T_RM_ similar in phenotype to those generated by influenza virus infection, providing long term, heterosubtypic protection, independent of circulating T cells and neutralising antibodies (70, 71). Tissue-resident alveolar macrophages have been found to limit CD8^+^ T_RM_ formation following murine influenza infection and may offer an attractive target for manipulation (72).

Intravascular, but not subcutaneous, administration of an agonistic anti-CD40 antibody alongside poly-IC : LC (a Toll-like receptor 3 activator) with HIV envelope peptide antigen directly stimulated APCs in the blood, MLN and lung to enhance pulmonary CD8^+^CD103^+^ T_RM_ formation in macaques (45). Intravenous BCG in macaques induces more antigen responsive pulmonary CD4^+^ and CD8^+^ T_RM_ than intradermal administration, with protection lasting 6 months later (73). Intratracheal boosting with BCG however following intradermal BCG vaccination enhances protection (74). Although this study only analysed peripheral blood to correlate increased CD4^+^ T_EM_ populations with improved protection, it is anticipated that local delivery of antigen to the lungs would also increase T_RM_/B_RM_ populations. Pulmonary mucosal BCG vaccination therefore offers superior protection against *Mtb* compared to standard intradermal vaccination (48, 75, 76).

VPM1002, a live BCG vaccine genetically modified to improve immunogenicity, outperforms live-attenuated BCG in preclinical testing and is undergoing clinical trials (NCT03152903) (77). Aerosol immunization with a mutated *Mtb* strain *Mtb*Δ*sigH* reduced bacterial burden and lung pathology when compared to aerosolised BCG following *Mtb* challenge in macaques (78). *Mtb*Δ*sigH* persisted for longer in the lungs than BCG and generated increased iBALT and CD69^+^ T cells in BAL, which likely include T_RM_. Since antigen is required for T_RM_/B_RM_ establishment, increasing its persistence enhances generation. Increasing antigen persistence using a cytomegalovirus vector encoding *Mtb* antigen inserts prevented disease in macaques through the establishment and maintenance of lung T_RM_ (79).

It is also possible that skin-resident T_RM_ generated through intradermal vaccination may enhance both local and systemic host responses to *Staphylococcus aureus*, a common commensal of the skin and nasal mucosa, to help minimise Staphylococcal pneumonia (80).

## Vaccination Strategies to Promote B_RM_

Strategies to induce pulmonary B_RM_ require delivery of antigen to the lung (7). In mice, intranasal vaccination extended antibody specificity to confer heterosubtypic protection by inducing GCs that generated cross-reactive antibody responses (14). In human, the squalene emulsion adjuvants AS03 or MF59 augmented neutralising antibody production when co-administered intramuscularly with influenza vaccine (81). Both adjuvants enhanced antigen uptake and presentation in local tissue leading to increased CD4^+^ and B cell responses, with AS03 also shown to increase naïve B cell activation and the adaptability of pre-existing memory B cells (82). Despite increasing the breadth of B cell repertoire following seasonal Flu vaccine (83), the impact of adjuvants on pulmonary T_RM_/B_RM_ remains to be demonstrated for such intramuscular vaccine. We could not exclude that a boost from an adjuvanted vaccine in humans previously exposed to a similar antigen encountered in the lung could re-activate and maintain pulmonary T_RM_/B_RM_.

In contrast, certain respiratory viruses such as RSV are known to trigger a T_H_2-like, dysregulated antiviral response (84). Acute RSV infection limits pulmonary B_RM_ formation (85) and encodes a number of immunomodulatory proteins that impair antigen presentation and type 1 interferon release (18), which may explain why infection is associated with a low level antibody response (21). Similarly, COVID-19 also suppresses MHC I/MHC II antigen presentation and interferon response (86). These issues of dysregulated T cell responses should be avoided or overcome through vaccination, leading to long-term humoral protection.

## Quantifying Immunological Memory Following Vaccination

Most vaccine studies in humans rely on peripheral blood sampling to evaluate protection. Serum haemagglutination inhibition (HAI), ELISA or ELISpot may indicate the humoral response generated against a given pathogen/vaccine, however does not always reflect immunity, as protection against influenza has been seen despite the absence of HAI titres (87). Nasal IgA is also a better reflector of protection to RSV than serum IgG (85). Circulating memory T and B cells do not always correlate with protection (8, 85) and immune responses can differ from those in lung (44).

Limited peripherally accessible biomarkers have been identified following immunization relating to resident memory lymphocyte generation. Early rises in plasma IL-10 correlated with pulmonary CD8^+^CD103^+^ T_RM_ generation following immunization in macaques (45). The CXCL10/CXCR3 axis has also been postulated as a potential biomarker for CD4^+^ migration to the lung (18). Further immunization studies correlating peripheral biomarkers with T_RM_/B_RM_ formation are required.

Airway T_RM_ can be isolated *via* BAL (30). Virus-specific CD8^+^ frequencies have been found to be 10 times higher in BAL than in peripheral blood in AGM, highlighting the quantitative differences between local and systemic T cell responses (21). BAL can be collected multiple times, providing temporal information on airway populations, but not interstitial. Post-mortem analysis is often the only method for assessing T_EM_/T_RM_. Tissue sections can be collected for histology or enzymatic tissue digestion, however accessing human/NHP tissue is difficult. Lung tissue is easily contaminated with alveolar/intravascular cells unless the organ is perfused and BAL collected (however this is not 100% effective). Intravenous antibody staining can distinguish tissue resident from circulatory cells, however, is not performed in humans/NHP. T_RM_/B_RM_ are identified through surface marker expression or gene signature, with pathogen-specificity evaluated through intracellular cytokine staining following exposure to antigen/MHC tetramers (T_RM_) or binding of labelled-antigen (B_RM_). Given the limited information gained on pulmonary T_RM_/B_RM_ populations using current sampling methods, new detection techniques are required.

### *In Situ* Optical Imaging

**Figure 3:**
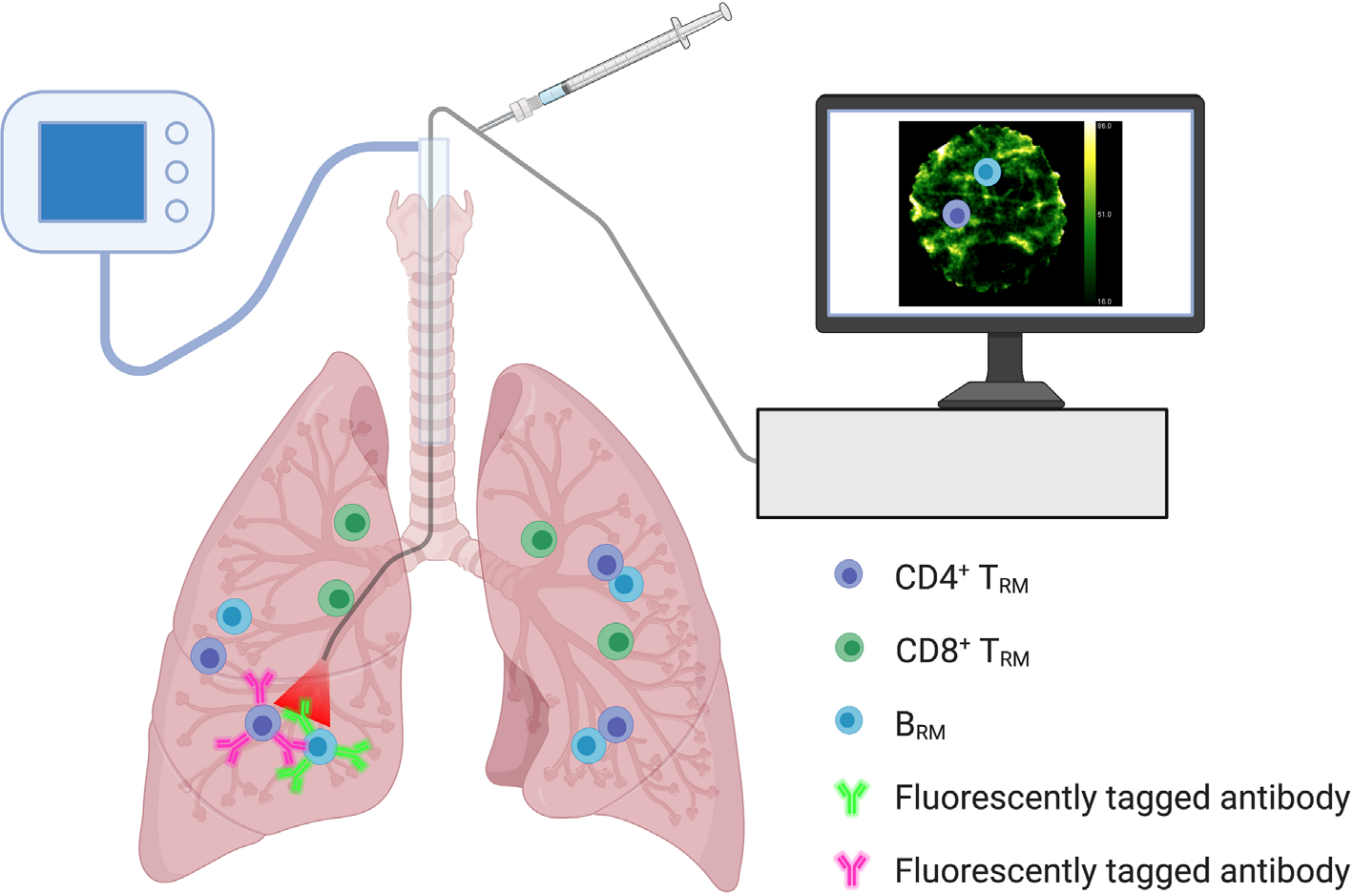
*In Situ* Optical Imaging of Resident Memory Lymphocytes. Optical endomicroscopy imaging within the lungs may allow for the *in situ* detection and quantification of resident memory lymphocyte populations. Monitoring numbers following immunization may help reflect vaccine efficacy and immunological memory. Fluorescently tagged ligands or antibodies, capable of binding to specific T_RM_/B_RM_ surface markers, can be delivered to the airways *via* a bronchoscope to enable visualisation. Using a combination of fluorescent ligands/antibodies could help differentiate resident memory lymphocyte populations.

Optical endomicroscopy imaging (88), recently used for the detection of human alveolar neutrophils *in situ* (89), may provide a valuable tool for assessing pulmonary-resident memory lymphocytes and quantifying immunological memory following vaccination. Fluorescently tagged ligands or antibodies, capable of binding to specific T_RM_/B_RM_ surface markers, can be delivered to the airways *via* a bronchoscope to enable visualisation (**Figure 3**). The information gained can be combined with systemic data to evaluate vaccine efficacy and expected degree of protection against respiratory pathogens. *In situ* optical imaging may also be used to screen lungs for transplantation, as the presence of T_RM_ in donor tissue is associated with reduced adverse clinical events in the recipient (30).

### *Ex Vivo* Lung Perfusion

The COVID-19 pandemic has highlighted how immune responses in the airways differ from those in the circulation and that it is tissues, not blood, where immune cells function (90). Assessing tissue-based immunity following infection and vaccination is therefore essential. *Ex vivo* lung perfusion (EVLP), using human lungs deemed non-suitable for transplantation, provides an ideal model for assessing tissue immunity and optimising *in situ* optical imaging. As well as studying populations *in situ*, EVLP offers the ability to isolate large numbers of human T_RM_/B_RM_, far higher than those obtained from a typical BAL, for in-depth analysis (including phenotype, function, and antigen-specificity). Intraperfusate delivery of a fluorescently tagged CD45 antibody can also differentiate circulating (labelled) from tissue-resident (non-labelled) cells. This technique has recently revealed how human lung T_RM_ colocalise with lung-resident macrophages, preferentially around the airways, where they receive costimulatory signals to augment effector cytokine production and degranulation (91).

## Concluding Remarks

Resident memory lymphocytes in the lung enhance immunity against respiratory pathogens. Understanding the mechanisms that drive T_RM_ and B_RM_ formation will improve vaccine design, with the hope of generating long lived, polyfunctional T_RM_ and broadly reactive, neutralising-antibody-secreting B_RM_ in the lung. Targeting respiratory APCs with antigen followed by subsequent “boosts” may establish and maintain these populations. Assessing the local and systemic responses using a combination of *in situ* imaging and peripheral blood sampling may reveal the efficacy of novel vaccines designed specifically to induce resident memory lymphocyte populations in the lung. Human *ex vivo* lung perfusion provides an ideal model for researching T_RM_/B_RM_ populations and optimising novel methods for their *in situ* detection to help quantify immunological memory.

## Author Contributions

DH, KD, MC-R, and VP contributed to conception and design of the manuscript. DH wrote the first draft of the manuscript. DH, RO’C, DL, MC-R, and VP wrote sections of the manuscript. All authors contributed to the article and approved the submitted version.

## Funding

This work was funded by Sanofi Pasteur.

## Conflict of Interest

KD is a founder and shareholder of Edinburgh Molecular Imaging. DH, DL, MC-R and VP were employed by Sanofi Pasteur.

The authors declare that this study received funding from Sanofi Pasteur. The funder had the following involvement in the study: study design, preparation of the manuscript and decision to publish.

The remaining author declares that the research was conducted in the absence of any commercial or financial relationships that could be construed as a potential conflict of interest.

## Publisher’s Note

All claims expressed in this article are solely those of the authors and do not necessarily represent those of their affiliated organizations, or those of the publisher, the editors and the reviewers. Any product that may be evaluated in this article, or claim that may be made by its manufacturer, is not guaranteed or endorsed by the publisher.
